# Assessment of the Adhesion to Decellularized Cardiac Patches of Stem Cells Through Single-Cell Force Spectroscopy (SCFS)

**DOI:** 10.3390/biomimetics11080520

**Published:** 2026-07-23

**Authors:** Rafael Daza, Marcos Vázquez, Raquel Tabraue-Rubio, Luis Colchero, Manuel Elices, Ricardo Sanz-Ruiz, Gustavo V. Guinea, Fivos Panetsos, José Pérez-Rigueiro, María Eugenia Fernández-Santos

**Affiliations:** 1 Departamento de Ciencia de Materiales, ETSI Caminos, Canales y Puertos, Universidad Politécnica de Madrid, 28040 Madrid, Spain; rafael.daza@upm.es (R.D.);; 2 Center for Biomedical Technology (CTB), Universidad Politécnica de Madrid, 28223 Pozuelo de Alarcón, Spain; 3 Department of Cardiology, Hospital General Universitario Gregorio Marañón and Instituto de Investigación Sanitaria Gregorio Marañón (IiSGM), 28007 Madrid, Spain; marcos.vazquez@iisgm.com (M.V.);; 4 Centro de Investigación Biomédica en Red—Enfermedades Cardiovasculares (CIBERCV), RICORS TERAV, ISCIII, 28029 Madrid, Spain; 5 Bioactive Surfaces S.L., C/ Puerto de Navacerrada 18, 28260 Galapagar, Spain; 6 Facultad de Medicina, Universidad Complutense de Madrid, 28040 Madrid, Spain; 7 Biomedical Research Networking Center in Bioengineering, Biomaterials and Nanomedicine (CIBER-BBN), 28029 Madrid, Spain; 8 Biomaterials and Regenerative Medicine Group, Instituto de Investigación Sanitaria del Hospital Clínico San Carlos (IdISSC), C/ Profesor Martín Lagos s/n, 28040 Madrid, Spain; 9 Neurocomputing and Neurorobotics Research Group, Faculty of Biology and Faculty of Optics, Universidad Complutense de Madrid, 28040 Madrid, Spain; 10 ATMPs Production Unit, IiS-Gregorio Marañón Health (IiSGM), Gregorio Marañón General University Hospital, 28007 Madrid, Spain

**Keywords:** single-cell force spectroscopy, decellularized matrix, mesenchymal cells, DeepTip^TM^ probes, atomic force microscopy

## Abstract

Cardiovascular diseases (CVDs) remain a leading source of morbidity and mortality worldwide. Although heart transplantation is an established treatment for selected patients with end-stage heart failure, the difficulty in obtaining enough donors and the problems associated with compatibility force the search for new strategies to mitigate these effects. Cardiac patches, a combination of therapeutic elements, such as cells and drugs deposited on a biomimetic scaffold, are particularly promising for repairing the long-term cardiac damage from a perspective based on tissue engineering. However, building these patches entails the compliance with extensive and exhaustive conditions and regulations. Key issues identified in the building process of cardiac patches are the cell–scaffold interaction, including the intensity and duration of this interaction. The present study is intended to develop a robust procedure that allows quantifying the adhesion between decellularized matrices and cells through single-cell force spectroscopy (SCFS) measurements. Determining this adhesion force is essential to ensure cell retention and therapeutic action in the damaged area and, therefore, to be able to design an advanced therapy medicinal product. The whole procedure is validated by measuring the adhesion of allogeneic adipose-derived mesenchymal stromal cells to a decellularized porcine myocardium patch.

## 1. Introduction

Cardiovascular diseases are the main cause of worldwide morbidity and responsible for over 17 million deaths annually [1]. Although surgical procedures may improve the cardiac function of the affected heart [1], a proper therapy that allows myocardial regeneration and remodeling is largely missing [2]. Consequently, new approaches based on cardiac tissue engineering have been proposed to improve the function of the damaged myocardium. These strategies are intended to recover heart function and to prevent ventricular remodeling in end-stage heart failure [3].

In this context, decellularized extracellular matrix (dECM) constitutes a promising biomimetic approach for repairing cardiovascular tissue, as dECM most effectively captures the complex array of proteins, glycosaminoglycans (GAGs), proteoglycans, and many other matrix components that are found in native tissue, providing ideal cues for regeneration, repair, and remodeling of damaged myocardium. dECM has been used in a variety of forms for cardiac therapy, described broadly as either solid scaffolds that maintain native vasculature and structure or soluble materials with the format of injectable hydrogels for tissue repair [3].

When compared with the direct injection of cells, the usage of cardiac patches as scaffolds allows the retention of a much larger number of cells that contribute to replace cardiomyocytes lost during the infarction [3,4,5,6]. Moreover, it is demonstrated that decellularized myocardial matrices, when recellularized using stem cells, can achieve high cell viability, homogeneous cell distribution, support cell growth, and promote cell infiltration within the scaffold [4,7]. Additionally, when the scaffolds were combined with adipose-derived stem cells, they induced the differentiation of part of the stem cell population into endothelial cells, developing a vascularized scaffold.

Combined with the material that constitutes the scaffold, the cellular component of the therapy plays a vital role in advanced tissue engineering therapies for MI treatment. Allogeneic adipose-derived mesenchymal stromal cells (AD-MSCs) have been shown to promote angiogenesis and are widely used in treating ischemic tissues. They present many benefits when used to treat cardiovascular diseases, such as their capability of differentiating into cardiomyocytes, vascular smooth muscle cells or endothelial cells. In addition, AD-MSCs show a potent paracrine effect that promotes neovascularization, reduces apoptosis, and inhibits fibrosis [4,8].

Since it was developed, atomic force microscopy [9] has been a tremendously versatile tool and has allowed atomic resolution images of a multitude of systems [10,11], as well as their manipulation [12]. An atomic force microscope (AFM) consists of a cantilever, usually with a sharp tip at its end, which is used to scan the surface of the sample of interest. Recently, the development of different techniques which can modify the chemical reactivity of the cantilevers has extended AFM capacities with the immobilization of either individual molecules or cells on the cantilever. In this way, it was possible to measure the force required to unfold a protein [13,14], to quantify the intensity of the antigen–antibody interaction [15], or to determine the strength of the adhesion between cells or between cells and solid substrates [16]. In this context, the functionalized DeepTip^TM^ probes have shown their adequacy for the measurement of even single-molecule recognition events [17,18,19].

In this work, a procedure that allows quantifying the interaction between cells and biomaterials is developed and validated through a model system that consists of decellularized cardiac matrices derived from porcine left ventricle and porcine AD-MSCs. In particular, the force between cells and matrix can be measured and the effect of various experimental parameters on its intensity explored. Since cell–scaffold interaction is critical for the development of a variety of biomimetic systems and, in particular, of those developed within the framework of tissue engineering, this procedure will probably become and essential tool for the design and implementation of these systems in the future.

## 2. Materials and Methods

### 2.1. Cell Culture

Porcine adipose-derived stem cells (pADSCs) sourced from the Cell Workbench for Cardiology Translational Research Laboratory (Madrid, Spain) were utilized. All porcine biological materials used in this study—specifically adipose tissue for pADSC isolation and cardiac tissue for myocardial decellularization—were harvested post-mortem immediately after euthanasia from donor swine. No live animal procedures were performed specifically for the purpose of this study. Several vials stored in liquid nitrogen at −196 °C were thawed, each vial containing 1 million cells in passage 4 suspended in 1 mL of cryoprotectant (CryoStor CS10 Merck, New York, NY, USA).

Cells were culture-expanded in 175 cm^2^ flasks, with DMEM (Gibco™, Thermo Fisher Scientific, Waltham, MA, USA) supplemented with 10% fetal bovine serum (FBS) and 1% penicillin–streptomycin (complete medium) to a total volume of 50 mL. Cells were cultured under standard conditions: 37 °C with 5% CO_2_ and 90% relative humidity. Twenty-four hours after thawing, the cell culture was washed with 15 mL of phosphate-buffered saline (PBS, Cytiva, Danaher Corporation, Washington, DC, USA) and the medium was replaced. Based on our experience, to optimize cell expansion, subsequent medium changes were performed every 48 h. The culture was expanded by cell passaging approximately every 6–7 days of culture, once they reached 80–90% confluency. At this point, the cells were detached with 15 mL of TrypLE Select (1X) (Gibco™), a recombinant enzyme replacement for trypsin, free of animal products, and incubated at 37 °C. After this time, they were collected and washed with DMEM and centrifuged at 300 g for 10 min at room temperature. The process was continued until the number of cells needed for the experiments was obtained: 10^5^ cells for the recellularization assays and 50,000 cells for the single-cell force spectroscopy (SCFS) assays.

### 2.2. Decellularized Matrix Preparation

The matrices were derived from 2 × 3 cm blocks of porcine myocardium, specifically from the left ventricle. Using a vibratome (7000smz-2 Campden Instruments, Loughborough, UK), sections were sliced into 500 or 900 µm thick sheets. At least five cuts were obtained from each block, where sixteen blocks from four different pigs were used. Hearts were obtained *post mortem* from pigs used for research purposes in the animal facility of Gregorio Marañón Hospital. The first cut was halved, freezing one part at −80 °C and decellularizing the other one for future DNA quantification and comparison. The second cut was also divided in half: one part was decellularized and subsequently immersed in formalin, while the other part was directly immersed in formalin for future histological analysis. The third, fourth, and fifth cuts were directly decellularized for subsequent applications in cell cultures or AFM assays.

The decellularization process was carried out by modifying our previously described protocols to remove the cells from the sheets while retaining the ECM [20]. Briefly, porcine left ventricle sections were decellularized with an anionic detergent, sodium dodecyl sulfate (SDS). This detergent disrupts the cell membranes of resident myocardial tissue cells, initiating cellular removal. The cuts designated for decellularization were immersed in 25–30 mL of 2% SDS for three days under agitation (Stuart™ Gyratory rocker, SSL3, Cole-Parmer, Vernon Hills, IL, USA) at room temperature, with SDS renewal twice a day. Then, the 2% SDS was replaced with 1% SDS for four days, renewing it once a day and, later, 1% SDS was replaced with 40–50 mL of distilled water for complete removal, and they were further agitated for an additional 96 h period under the same conditions. The second set of cuts were transferred into formalin after the decellularization period. Subsequently, all those decellularized matrices (dECMs) which were not immersed in formalin were washed with 20 mL of PBS (pH 7.0) supplemented with 1% penicillin–streptomycin (P-S). After washing, the dECM cuts were disinfected with an aqueous solution containing 1% peracetic acid for 30 min at room temperature with agitation. Then, they were transferred to sterile tubes containing 20 mL of PBS supplemented with antibiotics (1% P-S). After the washing period with distilled water, all next steps were carried out under entirely sterile conditions (Class II Biosafety Cabinet). The final containers were sealed and stored at 4 °C in the refrigerator until use.

### 2.3. Histological Analysis

The histological analysis of the matrices consisted of two stages: first, the samples were embedded in paraffin for subsequent sectioning and, then, they were stained to confirm the proper decellularization of the matrices.

Paraffin embedding was carried out using those samples previously fixed in buffered 3.7–4% formalin at a pH of 7 for a minimum of 6 h. Each paraffin block was sectioned using a rotary microtome (Microm HM 325 Thermo Scientific, Waltham, MA, USA). Sections of 5 µm thickness were mounted onto glass slides. Next, they were fixed at 90 °C in an oven for 30–40 min.

For staining, slices were prepared by removing paraffin excess in an oven at 60 °C for half an hour. Since multiple sections were obtained from each sample, one was allocated for hematoxylin and eosin (H&E) staining to detect cellular nuclei, while another was designated for Masson’s trichrome (MT) staining to check the maintenance of the structure based mainly on collagen. The remaining sections were stored. A Leica DM1000LED (Wetzlar, Germany) optical microscope was used for imaging.

### 2.4. DNA Quantification

Histology and DNA analysis were done in order to determine if our matrices essentially met the minimum characterization criteria based on detecting the removal of residual DNA responsible for most adverse host reactions [21,22]: absence of visible nucleated material in tissue sections stained with 40,6-dia-midino-2-phenylindole (DAPI) or hematoxylin and eosin (H&E) [23] (see previous section), <50 ng of dsDNA per mg dry weight of ECM, and DNA fragment length < 200 bp. DNA quantification was realized by means of a High Pure PCR Template Preparation Kit (Roche, Basel, Switzerland) following the manufacturer’s instructions. Briefly, 25–50 mg of tissue was incubated with a lysis solution for at least 1 h at 55 °C until tissue was completely digested. Then, the binding buffer was added and kept for 10 min at 70 °C and, later, the isopropanol was used to isolate DNA. Subsequently, the protocol for washing and elution was followed to obtain the deoxyribonucleic acids. A UV/Vis spectrophotometer (Implen NanoPhotometer 16, Implen, Munich, Germany) was employed to measure dsDNA concentration. No specific attempt was undertaken to determine the length of the small fraction of residual dsDNA.

### 2.5. Recellularization of Porcine dECM Sheets

Cell culturing on the porcine dECM sheets was performed on a multiwell plate with porous membranes of 0.4 μm pore size (Transwell™ Multiwell Plates with permeable polyester membrane inserts, Corning™, Corning, NY, USA), which allows the exchange of nutrients and medium but not the transfer of cells. The dECM sheets were exposed to UV light for 5 min. Then, 2 mL of culture medium was added to the bottom of each well of the plate before the dECM sheets were spread on the inserts. Subsequently 10^5^ cells labeled with a fluorescent cell membrane marker (CellTracker CM-DiI Dye, Thermo Fisher Scientific, Waltham, MA, USA) were added in each well, changing the medium every 48 h. Finally, the dECM sheets (matrices/patches) were fixed in formalin at different times: 72, 96, 120, 144 and 168 h, and kept in the fridge at 4 °C protected from light until imaging by inverted microscopy (Leica DMI3000B, Wetzlar, Germany) with the following observation parameters: exposition time 608.7 ms, gain 1 and gamma 0.8.

### 2.6. Matrices’ Topographical Characterization

The topographical characterization of the dECM was conducted using a Nanolife Atomic Force Microscope (Nanotec Electrónica S.L., Madrid, Spain) operating in jumping mode [24], and the data were analyzed employing the WSxM software (version 4.0) [25]. PNP-DB cantilevers (NanoWorld, Neuchâtel, Switzerland) with a nominal elastic constant of 0.06 Nm^−1^ and nominal resonance frequency of 17 kHz were used.

To assess matrices’ topography, a 500 μm thick patch was prepared and kept at 4 °C in PBS supplemented with penicillin–streptomycin until use. This patch was affixed to a 35 mm glass Petri dish (FluoroDish FD35PDL, World Precision Instruments, Sarasota, FL, USA) through a fibronectin coating prepared at 0.08% dilution in PBS and incubated for 24 h at 37 °C. After the coating was prepared, the patch was gently spread using tweezers on top of the Petri dish, allowing it to air-dry for two hours. When characterizing the patch, it was immersed in 2 mL of PBS for its natural physical recovery. The jumping mode was conducted under liquid conditions on the patch, scanning an area of 4.5 × 4.5 μm^2^ for each image, which were acquired at a resolution of 256 × 256 pixels. To prevent damage to the sample during its characterization, a limit value of force to be exerted was imposed: 0.5 nN.

### 2.7. AFM Cantilever Fluorescence Assays

DeepTip^TM^ SiN T17 tipless (Bioactive Surfaces S.L., Galapagar, Spain) were subjected to two different fluorescence assays. Firstly, cantilevers were cleaned with acetone, rinsed with distilled water, and dried with KIMTECH paper. Subsequently, they were incubated with 70 μL of biotin-NHS at 5.5 mg/mL in PBS for 30 min. For negative controls, biotin-NHS was replaced by PBS. Afterwards, chips were washed with PBS and 100 μL of PBS-buffered streptavidin–FITC (FITC-Streptavidin, Invitrogen™, Thermo Fisher Scientific, Waltham, MA, USA) were added to each chip for 30 min at room temperature and under dark conditions. Finally, cantilevers were washed with PBS and placed on a slide for analysis by fluorescence microscopy. The parameters used for the observation were as follows: exposition time 1 s, gain 2.1, gamma 0.83, and magnification × 20.

Alternatively, the AFM probes were subjected to the same initial steps of the previous procedure, but streptavidin–FITC was replaced by streptavidin 0.5 mg/mL in PBS and left in contact with the cantilevers for 30 min. Subsequently, 70 μL of biotinylated primary anti-CD29 antibody (ITGB1 Monoclonal Antibody (MEM-101A Biotin, Invitrogen, Thermo Fisher Scientific, Waltham, MA, USA)) diluted with PBS at different concentrations (10, 20, and 40 μg/mL) was added to the cantilevers for 1 h at room temperature. Finally, chips were washed with PBS and then incubated with 70 μL of secondary antibody conjugated with FITC (Goat anti-Mouse IgG (H + L) Secondary Antibody, FITC). This antibody was used at a 1:500 dilution in a 0.1% BSA buffer. Incubation was carried out for 1 h at room temperature in darkness. After washing with PBS, the chips were mounted on a microscope slide and imaged using fluorescence microscopy with the following observation parameters: exposition time 1 s, gain 2.1, gamma 0.83, and magnification × 20.

### 2.8. Cell–Matrix Adhesion Characterization

To measure the strength of cell adhesion to the matrices, a single cell was immobilized on the anti-CD29-antibody-decorated DeepTip^TM^ SiN T17 tipless probe. The probes were incubated for 30 min with 100 μL of biotin-NHS (Sulfo-NHS-LC-biotin EZ-Link™, Thermo Scientific™, Waltham, MA, USA) at a concentration of 5.5 mg/mL, subsequently washed with PBS, and lastly incubated with 70 μL of 0.5 mg/mL streptavidin for 30 min. The probes were then washed again with PBS and stored immersed in PBS at 4 °C for a maximum of one week. On the day of the cell–patch adhesion assay, the chip was dried with KIMTECH paper and incubated for 1 h with 70 μL of biotinylated anti-CD29 antibody (ITGB1 Monoclonal Antibody (MEM-101A) Biotin, Invitrogen, Thermo Fisher Scientific, Waltham, MA, USA) at a concentration of 20 μg/mL, diluted in PBS. Finally, it was washed with PBS before use. All steps were performed at room temperature.

Cell–matrix adhesion was measured with a Nanolife Atomic Force Microscope (Nanotec Electrónica, S.L., Madrid, Spain). The elastic constant of these cantilevers was assessed by means of the Sader method [26] supported by the WSxM software (version 4.0) [25]. After completing the calibration, the cantilever was immersed in PBS for approximately 1 h at room temperature (25 °C) to ensure thermal equilibrium and reduce thermal drift.

A 500 μm thick patch fragment was adhered to a 12 mm diameter circular coverslip using the minimum possible amount of tissue adhesive (Hystoacryl^®^ B. Braun, B. Braun Melsungen A.G., Melsungen, Gemany). The coverslip was then attached, using high vacuum grease (DOW CORNING^®^, Corning, NY, USA), to a 35 μm Petri dish (FluoroDish FD35PDL, World Precision Instruments, Sarasota, FL, USA), and 2 mL of filtered complete culture medium, DMEM, was added. Around 50,000 porcine adipose-derived mesenchymal cells sourced from the Cell Workbench for Cardiology Translational Research Laboratory were transferred to the Petri dish. The cantilever was positioned over the cell and slowly approached until a force of 100 pN was applied to the cell. This force was maintained constant for 10–15 s to allow the cell to adhere to the cantilever. Subsequently, after separating the cantilever–cell system from the surface, the cell was allowed to recover its basal state for 10–15 min. The successful adhesion of the cell to the anti-CD29-decorated cantilever was verified by slightly moving the cantilever away from the initial position. Adhesion assays were performed using the lithography mode of the WSxM software (version 4.0) [25], as it allows the user to design an experimental workflow customizing every approach/retraction step. Each SCFS assay consists of a series of approach–retraction cycles between the cell attached to the cantilever and the patch or between the cell attached and the coverslip used as control substrate. Each cycle consists of 4 steps. (1) Forward movement, in which the cell approaches the matrix at a speed of 200 nm/s until a force of 100 pN is reached. (2) Contact between the cell and matrix for a period of either 15 s or of 30 s (contact time). (3) Retraction of the cell from the matrix at a constant speed. Speeds of 36 μm/s, 3.6 μm/s or 0.36 μm/s were used in different experiments. (4) The cell is allowed to recover for 60 s before proceeding to the next cycle. For each pair of conditions defined by contact time and retraction speed, five cycles were performed, so each cell was subjected to 30 cycles. Seven experimental sessions were conducted, of which only five yielded satisfactory results due to cell immobilization issues or patch collisions of the other two. Consequently, five independent cells were analyzed. Each cell was immobilized on a different cantilever and tested against a distinct matrix.

The data were obtained as raw text files, containing the deflection values (V) at each height of the piezoelectric system (nm) for every approach–retraction cycle. Through prior calibration of the cantilever, the deflection measurements of the cantilever–cell system were converted to force units (nN). Two quantitative parameters describing the adhesion between the cell and the matrix were extracted: the separation or detachment force (F_d_) and the effective spring constant (K_s_). For this purpose, a MATLAB routine was used to automatically analyse each curve, obtaining both parameters while minimizing the effects of hydrodynamic forces and thermal drift.. To mitigate the effects of thermal drift and hydrodynamic drag in SCFS, the standard approach involves rotating and correcting the baseline of the force–displacement curves by applying a linear fit to the non-contact region and subtracting this mathematical slope from the entire curve [27]. To perform this correction, the routine selected the region of the retraction curve that was completely removed from the final rupture event. A linear regression (least squares) was then applied exclusively to the data points within this selected non-contact region, thereby obtaining the mathematical expression of the spurious baseline. Finally, for each point of the entire curve, the routine subtracted the drift component to force the baseline to be horizontal and yield a net force value of zero. Mathematically, this process is equivalent to an adapted matrix rotation, aligning the force axis with the displacement axis of the piezoelectric element. Once the baseline was corrected, the MATLAB (v 24.1) routine performed two additional processes. First, it determined the minimum recorded force value, which was defined as the detachment force. Second, it performed a linear fit to the force data immediately following the minimum, corresponding to increasing values of piezo displacement. The slope of this linear fit yielded the effective spring constant.

## 3. Results

### 3.1. dECM Sheet Preparation and Histological Characterization

**Figure 1:**
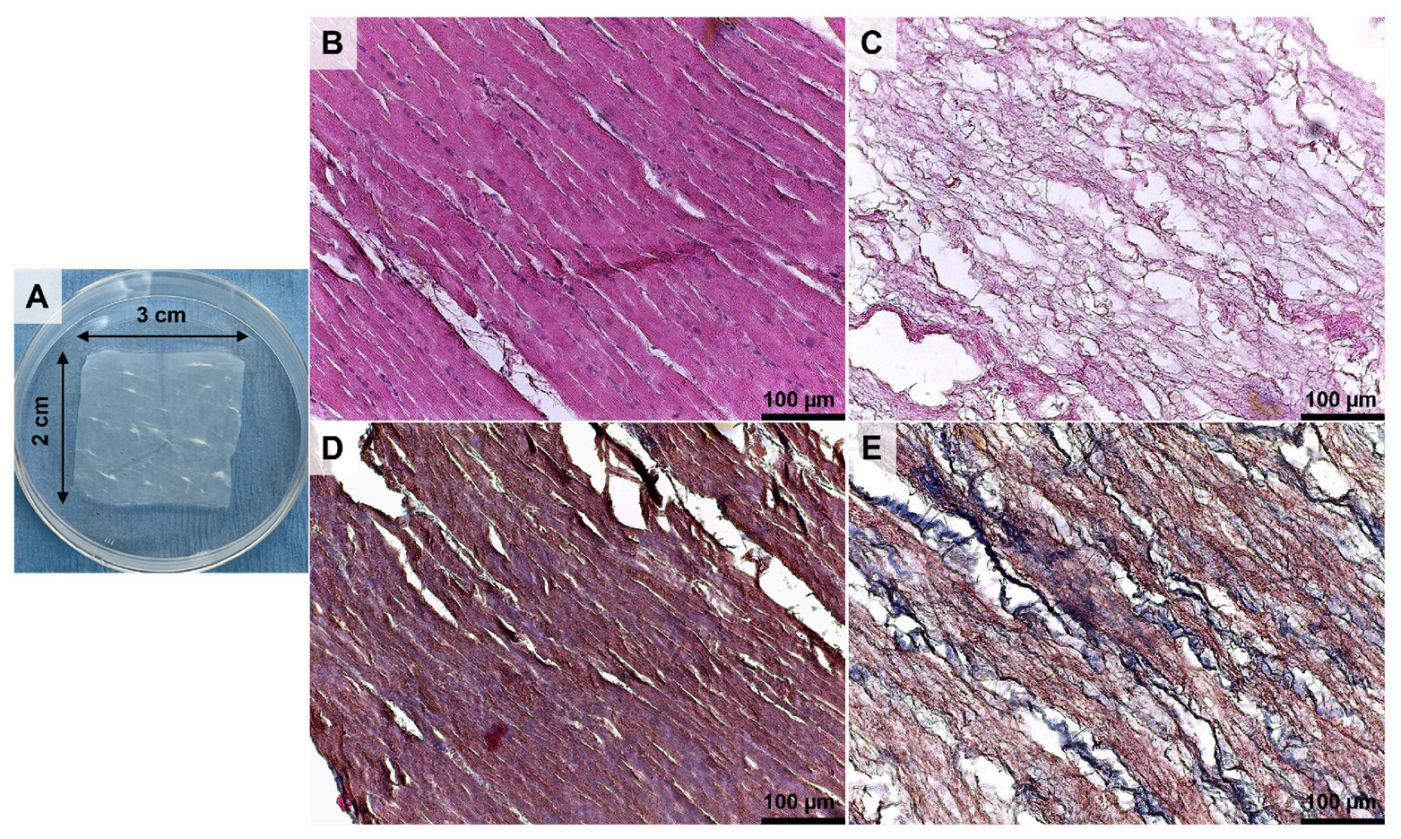
Histological analyses of the cardiac patches: (**A**) 2 × 3 cm cardiac patch. (**B**) H&E staining of control tissue shows cellular nuclei (blue) and collagen fibers (pink). (**C**) TM staining of the control tissue exhibits collagen fibers (blue), cytoplasm (red), and cellular nuclei (yellow/brown). (**D**) H&E staining of the decellularized matrix shows the absence of cellular nuclei, while preserving collagen fibers (pink). (**E**) TM staining shows that the decellularized matrix retains collagen fibers (blue).

Representative images of the dECM sheets are shown in Figure 1. The sheets have an approximately rectangular shape with an estimated area of 6 cm^2^ (Figure 1A). To verify that the decellularization process was successful, histological analyses were realized on both native tissue from porcine myocardium (control) and on decellularized sheets. For the detection of nuclei, the sheets were stained with H&E. Additionally, Masson’s trichrome was used for the identification of collagen fibers of the control and dECM sheets. The results confirm the presence of collagen fibers in non-decellularized samples but also demonstrate the preservation of a characteristic structure in the dECM. After the decellularization process, the dyeing with H&E reveals the absence of cells and the presence of collagen. Additionally, Masson’s trichrome staining evidences that the structure of the extracellular matrix remains intact after treatment with sodium dodecyl sulfate in the decellularized cardiac tissue.

### 3.2. DNA Quantification

**Figure 2:**
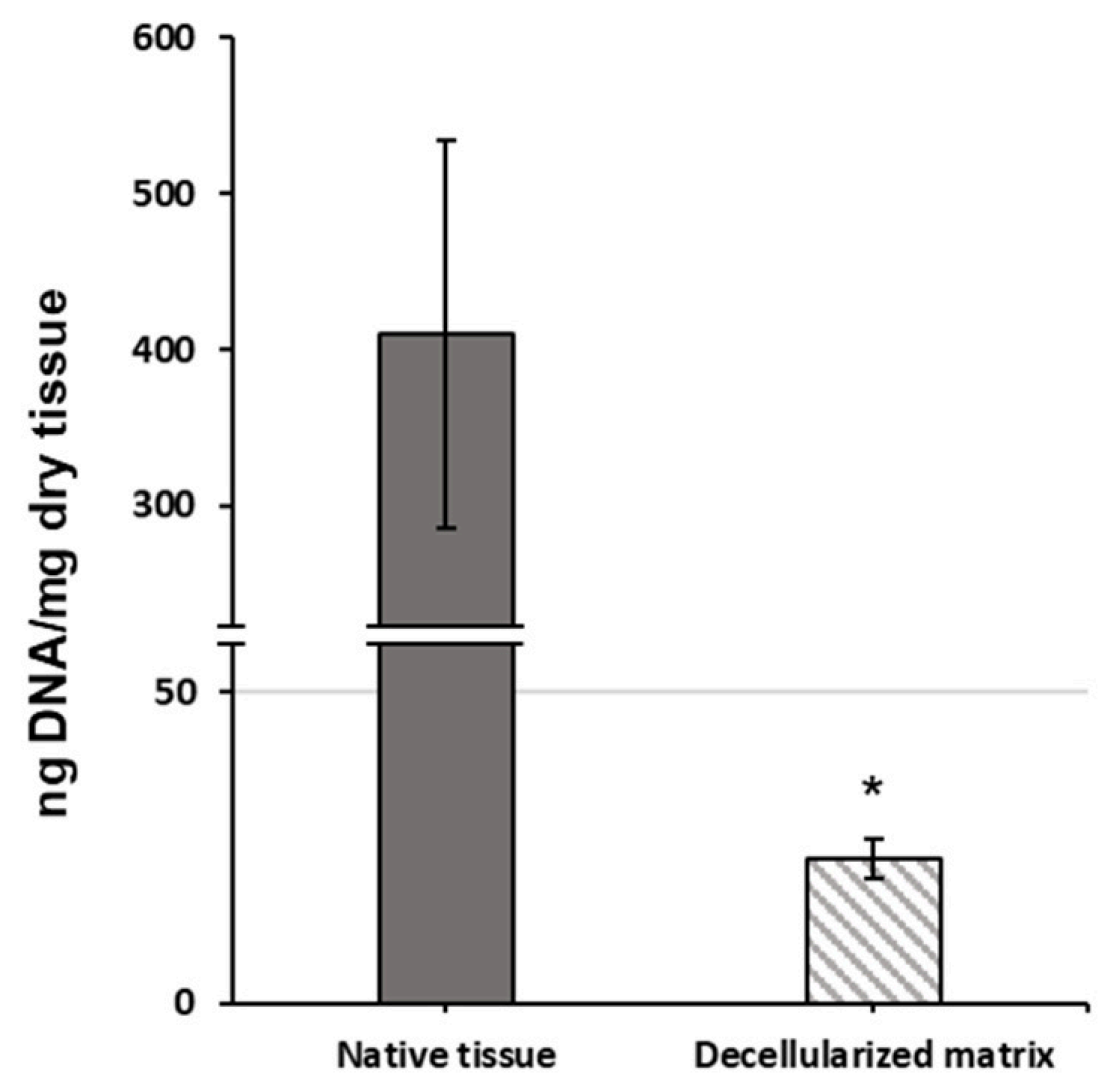
Comparison of DNA concentration between native cardiac tissue (control) and decellularized extracellular matrix (dECM) sheets. DNA concentration expressed a mean ± SE of 400 ± 100 and 23 ± 3 ng of DNA/per mg of dry weight tissue in control samples and dECM respectively. * *p* < 0.05 (Student’s *t*-test). (*n* = 3 for each group).

DNA quantification allowed us to verify, together with the histology shown in the previous section, that the decellularization protocol worked to obtain a correct decellularization of the matrices, fulfilling two of the minimum criteria established by Crapo [23]. The 11-day incubation protocol, based simply on the use of SDS and distilled water, achieved properly decellularized matrices with a DNA concentration of less than 50 ng/mg of dry tissue (Figure 2).

### 3.3. Recellularization of dECM

**Figure 3:**
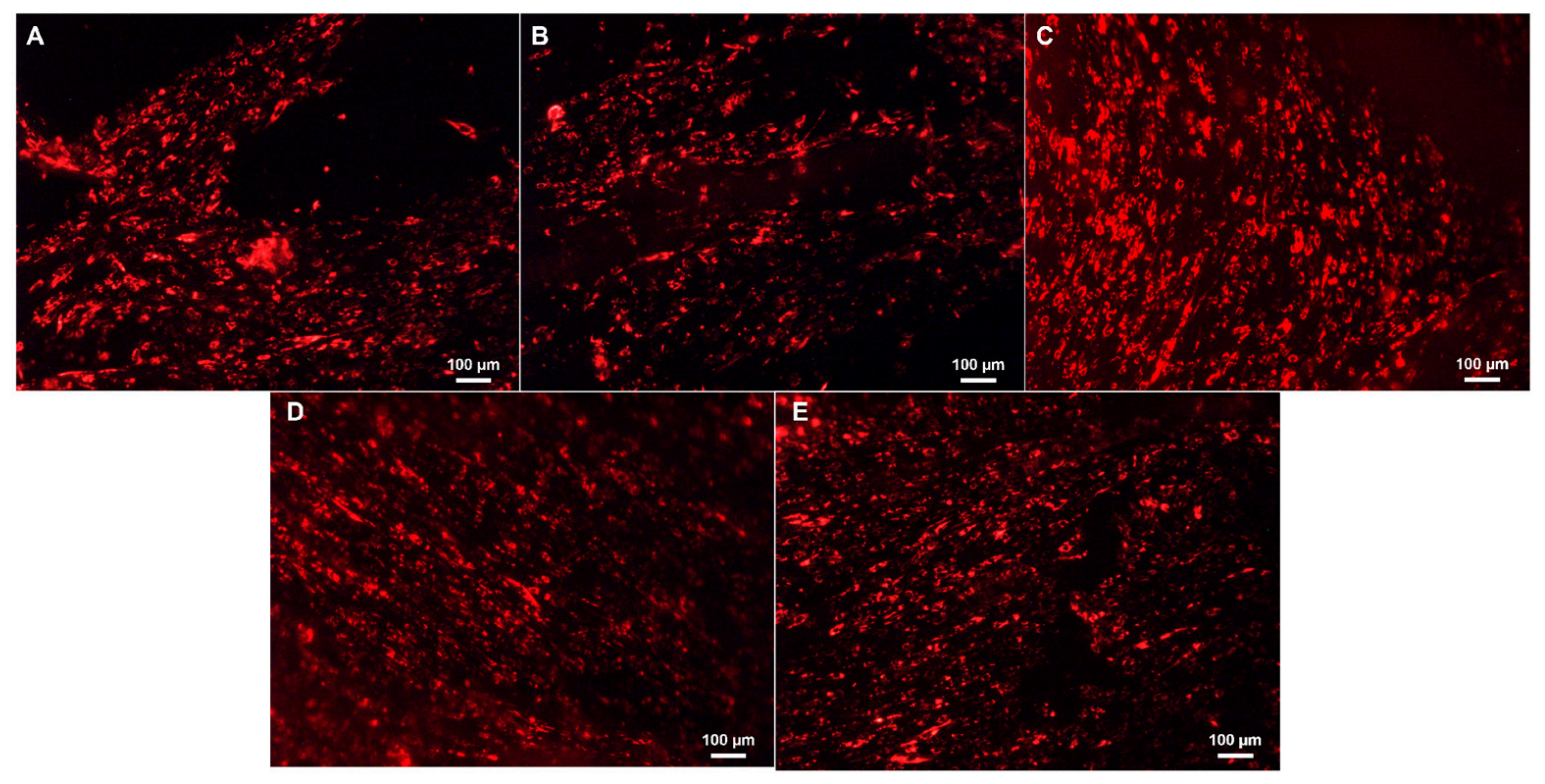
Cells labeled with DiI Dye on cardiac patch. Images were captured at 72 h (**A**), 96 h (**B**), 120 h (**C**), 144 h (**D**), and 168 h (**E**) (initial cell density = 10^5^ cells).

CellTracker™ CM-DiI Dye was used to characterize the behavior of living cells on the matrices in vitro. Mesenchymal stem cells were properly attached to the patch 72 h after being seeded (Figure 3). Since this dye remains in the cell membrane for several days, it was possible to assess its persistence and, therefore, cellular distribution on the matrices. In this regard, it was observed that the cells remained attached, exhibiting normal cell morphology and covering practically the entire surface of the matrices throughout the assay period, up to 7 days (Figure 3B–E).

### 3.4. Topographical Characterization of Decellularized Sheets

**Figure 4:**
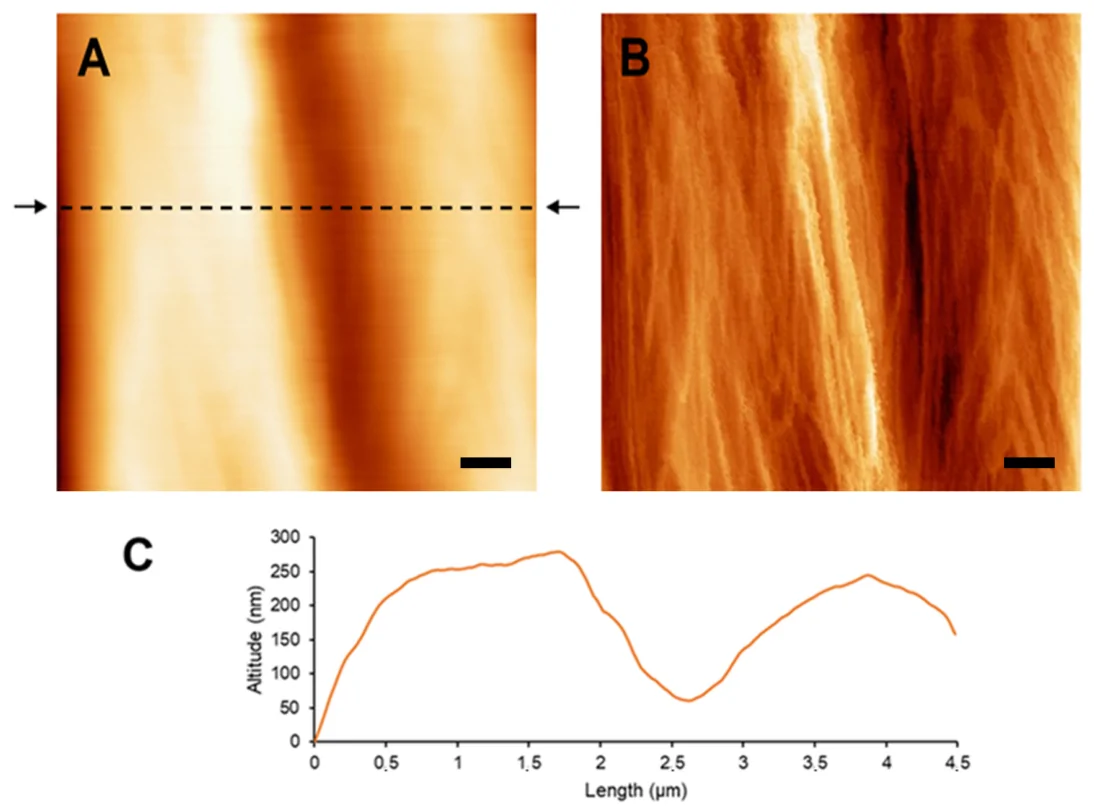
(**A**) Surface topography of cardiac patch (scale bar = 500 nm). (**B**) Error signal of image 4A, showing the characteristic fibrillar structure of collagenous extracellular matrices. (**C**) Height profile along the broken line limited by the arrows of Figure 4A.

A Nanolife AFM (Nanotec Electrónica S.L.) was employed to characterize the surface of the patches in jumping mode [24]. Figure 4A shows a representative topography image of the dECM sheet. It is observed that the surface of the matrix presents a roughness in the range of few hundreds of nanometers, well below the typical size of cells, in the range of a few microns. In addition, the preservation of the original organization of the dECM is verified from the recording of the signal error of the image as shown in Figure 4B. The existence of a typical collagen fibrillar structure is apparent in this micrograph, as expected from the alignment of the collagen fibers that make up the matrix scaffold. The profile measured along a straight trajectory in Figure 4A is presented in Figure 4C.

### 3.5. AFM Cantilever Fluorescence Assays

**Figure 5:**
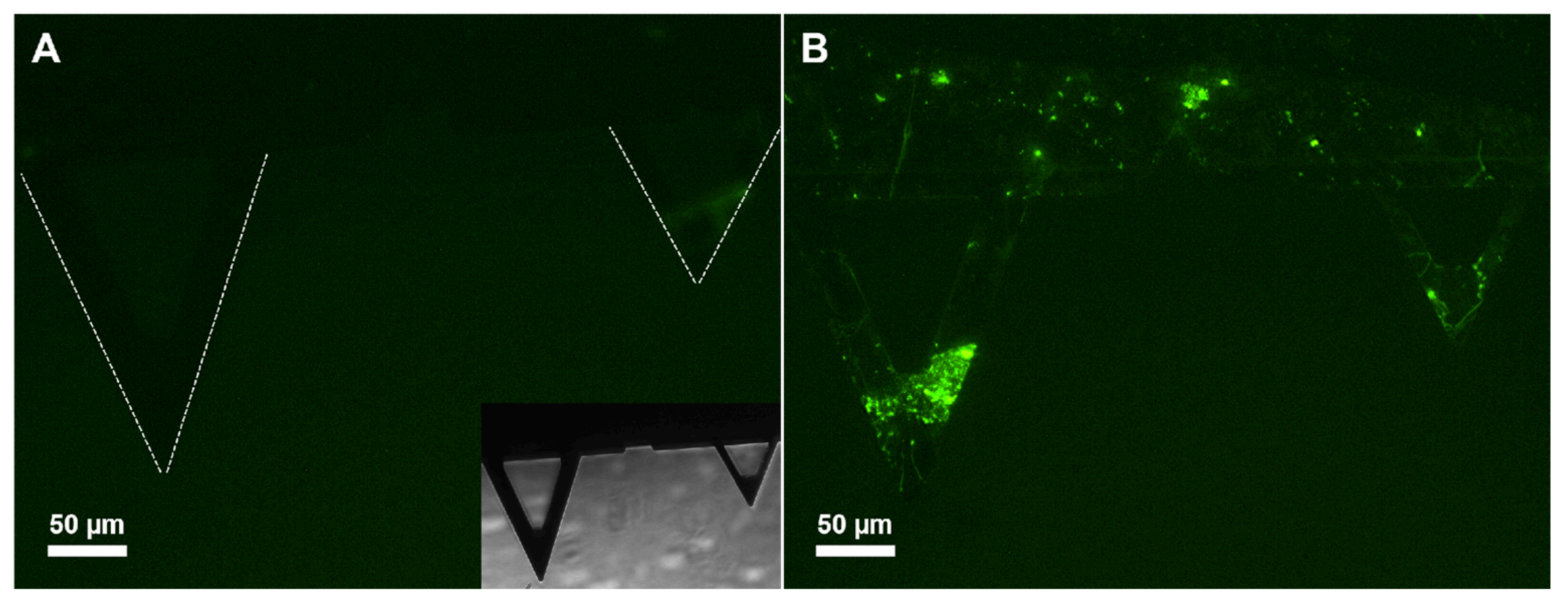
Fluorescence microscopy images of DeepTip^TM^ probes. (**A**) Negative control. The cantilevers are silhouetted to allow their identification, and the inset shows the light microscopy image of the cantilevers. (**B**) DeepTip^TM^ probe incubated with biotin-NHS.

The ability of the DeepTip^TM^ probe to bind biotin-NHS was assessed through fluorescence microscopy. Figure 5 compares the fluorescence observed in a negative control in which the DeepTip^TM^ probe incubation with NHS-biotin was replaced by simple incubation with PBS (Figure 5A) and a sample incubated with this molecule (Figure 5B). Both samples were subsequently immersed in an FITC–streptavidin solution.

The chemical modification of the probe through biotin is successful (Figure 5B), as observed from the higher intensity of the fluorescence signal compared with the control. Both micrographs, however, show a relatively inhomogeneous distribution of the fluorescence.

**Figure 6:**
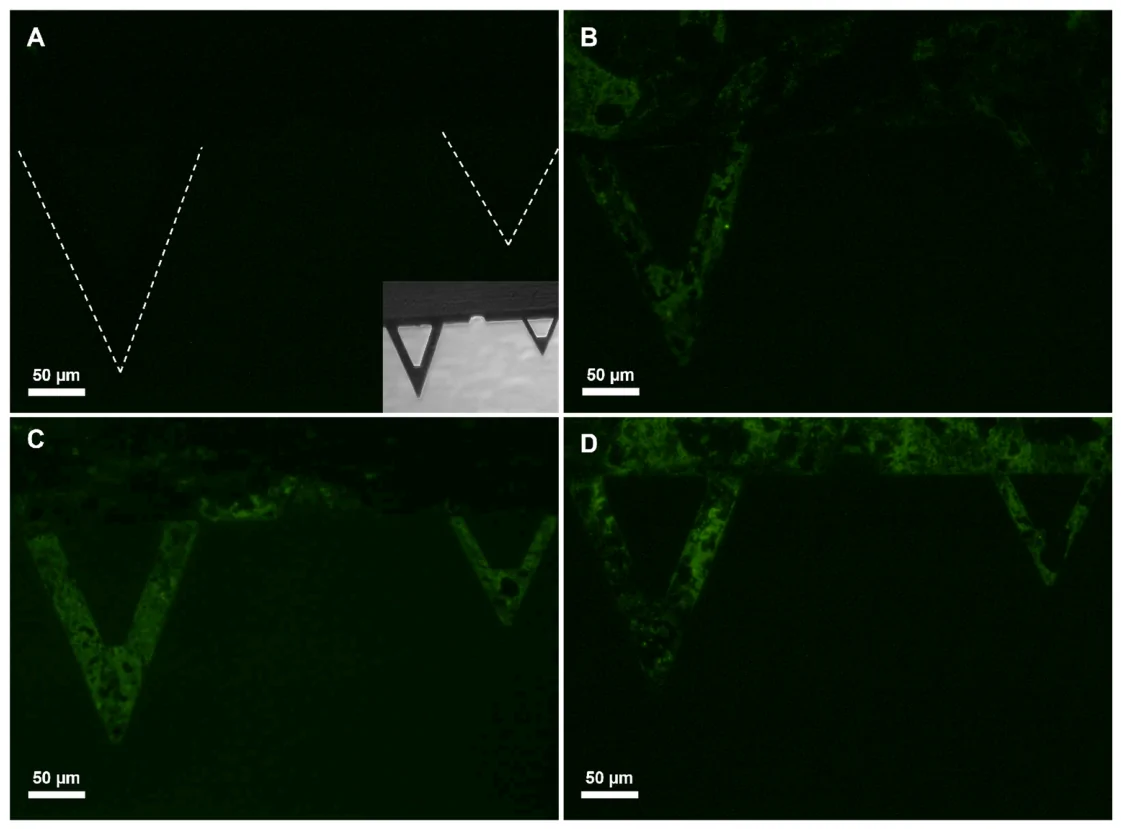
Fluorescence microscopy images of DeepTip^TM^ probes decorated with anti-CD29 antibodies. (**A**) Negative control without primary antibody. The cantilevers are silhouetted to allow their identification and the inset shows the light microscopy image of the cantilevers. (**B**) Probe decorated using a 10 μg/mL solution of anti-CD29 primary antibody. (**C**) Probe decorated using a 20 μg/mL solution of anti-CD29 primary antibody. (**D**) Probe decorated using a 40 μg/mL solution of anti-CD29 primary antibody.

An additional fluorescence assay was performed to establish the optimal antibody concentration to be used for decorating the cantilevers, and the result is presented in Figure 6. As can be observed, no fluorescence is detected in the non-decorated (control) cantilevers (Figure 6A). In contrast, cantilevers decorated with 10 µg/mL of biotinylated anti-CD29 antibody exhibit significant fluorescence, although with an inhomogeneous distribution (Figure 6B). A more homogeneous distribution of fluorescence in the chip and in the cantilevers is observed in the samples incubated with concentrations 20 µg/mL (Figure 6C) and 40 µg/mL (Figure 6D). The highest homogeneity observed with the 20 µg/mL solution led to its selection for decorating the probes used in the force–displacement measurements.

### 3.6. Cell–Matrix Adhesion Characterization

**Figure 7:**
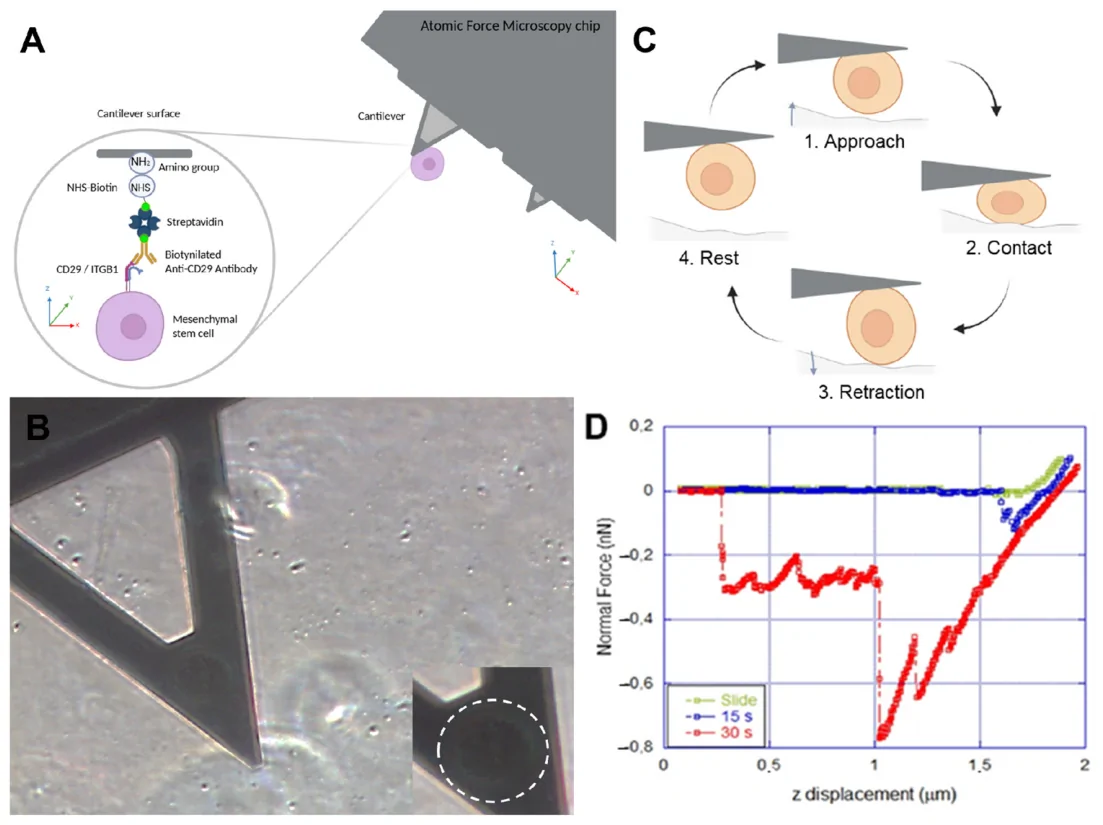
Workflow of a single-cell force spectroscopy experiment. (**A**) Decoration of the DeepTip^TM^ probes with anti-CD29 antibodies Created by BioRender. (**B**) Light microscopy micrograph of a porcine adipose-derived mesenchymal stem cell attached to the cantilever. The border of the cell is silhouetted. (**C**) The four steps of an SCFS experiment. (**D**) Illustrative force–displacement curves obtained during the retraction step after a latency time of contact between the cell and the cardiac patch of either 15 or 30 s. A retraction curve obtained after a contact time of 30 s between the cell and a control substrate is indicated in the figure legend as “slide”. (positive force: compression; positive displacement: approaching).

Single-cell force spectroscopy (SCFS) was used to analyze the early adhesion events between cells and cardiac patches. In SCFS, a cell is first attached to the cantilever and, subsequently, brought into contact with the surface to be analyzed. After a latency contact time, the cell–cantilever system is retracted, and adhesion events are recorded as variations in the deflection of the cantilever, as registered in the retraction force–distance curve. A critical step in this procedure is the immobilization of the cell on the probe, since if the attachment of the cell to the cantilever is not intense enough, the cell will be detached from the cantilever upon contacting the surface, rendering impossible the measurement of the adhesion force. To avoid this major drawback, a robust and versatile cantilever functionalization procedure was developed for this work that ends up with the decoration of the probe with an anti-CD29 antibody, as summarized in Figure 7A. A micrograph of the cell fixed to the decorated cantilever is presented in Figure 7B, in which the border of the cell is silhouetted with a broken line. The four different steps carried out to measure the adhesion forces between a cell and a substrate are indicated schematically in Figure 7C. In SCFS the main information is obtained in the fourth and last step (retraction) since it corresponds to the evolution of the cantilever deflection when the cell–cantilever system is moved away from the substrate. Three illustrative force–displacement curves obtained during the retraction step are depicted in Figure 7D: one of them was measured with the cell–cantilever system being in contact with a glass substrate (control substrate), whereas the other two force–distance curves were recorded when the cell remained in contact with the cardiac patch for either 15 or 30 s, respectively.

**Figure 8:**
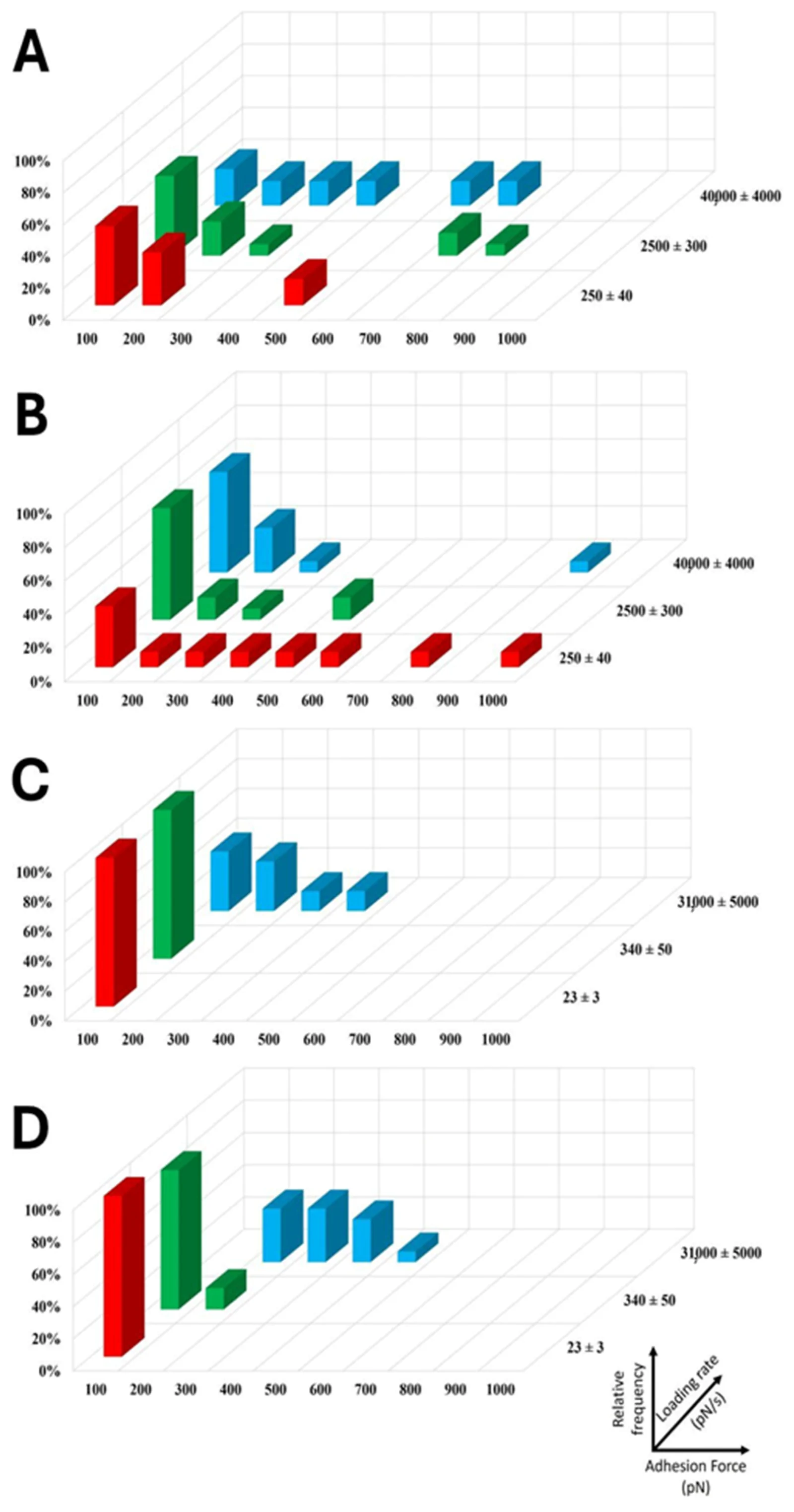
Adhesion forces as a function of the retraction speed after a contact time with the cardiac patches of either 15 s (**A**) or 30 s (**B**), and with the control substrates after a contact time of 15 s (**C**) or of 30 s (**D**).

Single-cell force spectroscopy was performed not only with different contact times but also with different retraction speeds. This is equivalent to analyzing the adhesion forces between cell and substrate at different loading rates and, therefore, it contains information on the specific or unspecific character of the interaction between cell and substrate. Retraction velocities were converted into loading rates by multiplying the velocity by the effective spring constant (K_s_), which was obtained from the slope of the linear relationship between the deflection of the cantilever and piezo displacement near the detachment point as explained above. This experimental approach may be used to model the interaction between cell and substrate [28] but, since the main scope of this work is the development of a robust and versatile SCFS methodology for the assessment of the interaction between cells and cardiac patches, this procedure will not be pursued further here. Figure 8 shows the histograms of the measurements of interaction events between the cell and the substrate as a function of the loading rates in the four experimental conditions explored: cells kept in contact with the patches for 15 s (Figure 8A) or for 30 s (Figure 8B), and cells kept in contact with the control substrates for both times (Figure 8C, latency time: 15 s and Figure 8D, latency time: 30 s).

## 4. Discussion

Cardiovascular diseases (CVDs) remain a leading source of morbidity and mortality worldwide [29]. Ischemic heart disease is one of the most frequent causes of heart failure (HF) and it is normally attributed to coronary artery disease, defined by the presence of one or more obstructive plaques, which provoke a reduced coronary blood flow, causing myocardial ischemia and consequent heart failure [30]. HF is a clinical syndrome characterized by dyspnea or exertional limitation due to impairment of ventricular filling or ejection of blood or both. In left ventricular HF, the left side must work harder to pump the same amount of blood than a healthy heart, with an increased demand between 55 and 70 percent. Once developed, heart failure results in significant morbidity and mortality, with a 1-year mortality rate of 7.2% and a 1-year hospitalization rate of 31.9% in patients with chronic heart failure. In patients hospitalized for acute heart failure, these rates increase to 17.4% and 43.9% [31,32].

Since cardiac regeneration is limited in adults, damaged cardiac regions form compensatory scars with very few functional cardiomyocytes, ultimately resulting in cardiac dysfunction and chronic heart failure. Current clinical therapies have been shown to enhance cardiac function, but none of them is designed to directly address the restoration of cardiomyocyte loss [33]. In recent years, the development of tissue engineering and regenerative medicine has opened new approaches to this problem, with cardiac patches being one of the most promising therapeutic approaches [34]. Cardiac patch technology is composed of a combination of cells, molecules and a scaffold [35], which implies the existence of several possible alternatives that, in addition, are constantly growing. Thus, different cell types such as myoblasts or stem cells are considered for their therapeutic capabilities, in combination with various bioactive molecules, such as growth factors or extracellular vesicles. With regard to the scaffold that supports both cells and bioactive molecules, two main groups can be distinguished: synthetic (artificial), mainly of polymeric origin, and biomimetic, mostly of natural origin. Decellularized matrices, portions of tissues subjected to different physicochemical treatments to eliminate the cells they originally contain, are an important example of the latter. Primarily, but not exclusively, from cardiac sources such as pericardium or myocardium, decellularized matrices provide an ideal environment to promote cardiac tissue restoration. However, the preparation of these decellularized matrices for use as a cardiac patch presents a great number of difficulties, one of the most notable being the loss of the structure necessary to support new cells.

In addition to the previous discussion, and when the clinical treatment of heart diseases is considered, rapid intervention in the immediate aftermath of heart failure appears as a critical condition that is crucial for the patient’s future. In this sense, it is a priority that cardiac patches can be implanted in the damaged area of the heart as quickly as possible. The demand to produce cardiac patches that may be employed as an off-the-shelf product is therefore especially pressing. This issue imposes even more severe limitations on the development of the cardiac patches than those mentioned above. To begin with, the origin of the cells employed for the therapy should be carefully considered. Although the EMA allows the usage of either autologous or allogeneic cells, the necessary immediacy of response after heart failure practically imposes the option of an allogeneic origin. Furthermore, although allogeneic cells can generate some type of response from the immune system of the patient, as established by Joyce et al. [36], there are multiple advantages to their use when producing an off-the-shelf product, mainly those that derive from the existence of master cell banks where cells proliferate and are maintained under optimal conditions, which limits their variability. However, there is no consensus on which cell types are the most suitable for implantation in a cardiac patch [33]. Early studies in this field suggested that bone marrow cells, bone-marrow-purified hematopoietic stem cells (HSCs), and bone-marrow-purified mesenchymal stem cells (MSCs) can differentiate into cardiomyocytes. Therefore, they would seem to be the main candidates for implantation. However, different laboratories proved that the intended differentiation into cardiac tissue did not take place or it is not as successful as required [37,38]. Even so, several theories indicate that using these types of cells together with various molecules, such as growth factors [39,40] or exosomes [41], could enhance cell differentiation. Another promising line of research contemplates the possibility of using embryonic stem cells and pluripotent stem cells for obtaining cardiomyocytes [42]. Although with these therapies remuscularization of the heart is observed and, therefore, a certain recovery of cardiac function is achieved, it has been found that their use is associated with the appearance of arrhythmias which, in some cases, may be fatal, and, in general, leads to the obtaining of cardiomyocytes with a high degree of immaturity. Once again, research is aimed at the combined use, on the selected scaffold, of cell types and drugs that may prevent arrhythmias and promote cardiomyocyte maturation. In conclusion, and despite the enormous amount of research being carried out in the field of cardiac regeneration regarding cardiac patches, the ideal combination of cell type, growth factors and/or drugs, and scaffold that results in a remarkable recovery of cardiac function remains an open question.

Additionally, and with regard to the procedures followed to produce cardiac patches in order to consider them as an off-the-shelf product, another major limitation is imposed by the need to be provided as fresh rather than as frozen material. This has important consequences since: (1) the maximum storage time of the patch before implantation rarely exceeds 72 h [36], being estimated that, after the fourth day, the cells begin to deteriorate and lose their function, and (2) cells must be cultured on the scaffold only when the patch is about to be implanted, remaining in suspension until that time, which requires adequate levels of oxygenation and nutrition. This issue is perhaps responsible for one of the major problems of this cell therapy: engraftment, i.e., the rapid decrease in the number of cells remaining in the patch a few hours after it is implanted in the patient [43]. There are many theories about the circumstances that cause this low cellular retention, reflected in the proposal of various strategies to overcome it. Among them, one of the most promising strategies is to deliver therapeutic cells in a cardiac patch sutured onto the heart surface [44,45,46]. Since this procedure requires open-chest surgery, in principle, it is only indicated for patients with severe heart failure, unless the benefit/risk ratio is very high, in which case this practice could be extended to other less acute cases. However, given the multitude of combinations of elements to be implanted (different cell types, different growth factors and/or drugs that favor cell survival, and different scaffolds), and the need to assemble all of them a few hours before the patch is to be implanted in the patient, it is very complicated to comply with all these requirements optimally.

In this context, the development of a procedure that allows the assessment of the interaction between cells and scaffolds within this large number of alternatives constitutes an essential tool for the obtaining of efficient cardiac patches. Such a procedure is presented in this work and their constituent elements discussed in detail. First, the efficiency of the decellularization process on sheets derived from porcine myocardium is validated. It is further shown that dECMs preserve their native collagen-rich structure and maintain their ability to anchor cells. In this regard, cytotoxic effects resulting from the decellularization process are precluded and cells proliferate until reaching confluence in approximately one week.

The characterization of the sheet is complemented with a topographical analysis using AFM that further confirms the presence of the original fibrillar organization at a submicrometric scale. This fibrillar organization is especially apparent from the representation of the error sign parameter of AFM images. In addition, for the subsequent SCFS tests, topography AFM micrographs confirmed that there were no large amounts of unevenness or irregularities on the surface that could lead to artefacts in the AFM measurements to be performed.

Lastly, it is shown how SCFS constitutes a robust methodology that allows obtaining precise quantitative measurements of early adhesion events in the model system constituted by porcine adipose-derived mesenchymal cells and decellularized matrix presented in this work. A detailed protocol that allows the decoration of the functionalized DeepTip^TM^ probes with anti-CD29 antibody is presented, taking advantage of the presence of the protein CD29, an adhesion receptor and co-stimulatory molecule, on the membrane of the mesenchymal cells. The usage of an integrin as the adhesion motif in the cell that allows the attachment to the cantilever opens the question of the possible effect that the clustering of integrins [47] might have on the effectiveness of the whole scheme. Although no specific experiments were performed to assess this question, the ability of antibody-decorated DeepTip^TM^ AFM probes to detect a single molecule interaction between an antibody and its target molecule [18] suggests that the procedure presented above may be largely independent of this aspect of the organization of the integrins in the cell membrane.

The performance of the SCFS experiments requires establishing two critical parameters: contact (or latency) time and retraction speed. Two different contact times were evaluated: 15 s and 30 s. In addition, control tests were performed in which the adhesion of the cell to the glass substrate (control) was measured. It is found that, when cells are put in contact with the control glass slide, even for the highest contact times, no relevant adhesion forces are measured. Comparatively larger forces are measured when the cell is placed in contact with the decellularized matrix, with both the complexity and intensity of these forces escalating with prolonged contact time. The recording of the cell–matrix interaction with this level of detail should be highlighted, since one of the major problems of the strategies followed to date is the fact that the cell population that remains attached to the patch represents just a small percentage of the total number of initial cells. It may be assumed that a larger number of interaction events of high intensity will result in a greater stability of the cells on the patch, so that their assessment could be correlated with the persistence of the cells on the scaffold upon implantation.

It is also found that there is an important dependence of the quantitative measured values of the adhesion forces on the retraction speed during the last step of the SCFS experiment. As can be observed, at low and medium retraction speeds, a proportion of events close to 50% show low adhesion forces (below 100 pN). These values of the adhesion force can be considered as mainly resulting from non-specific interaction [19]. It is also observed how adhesion forces increase at the highest retraction speed, both on the controls and on the patches. This dependence of the adhesion forces with the retraction speed has been reported previously [48,49,50] and assigned to two possible effects or to their combination: (i) a hardening process undergone by the interaction between cell and substrate at increasing strain rates, and (ii) the activation of a mechanism at the molecular level inside the cell that leads to an increase in its adhesion to the material, in which the protein talin might be involved [51]. In addition, it cannot be discarded that hydrodynamic effects related to the fast movement of the cantilever in a liquid medium might also be reflected as an increase in the recorded adhesion forces. Janovjak et al. [50] quantified the influence of these artefacts in single-molecule force spectroscopy measurements and established that the contribution of these hydrodynamic effects to the force–displacement curves are of the order of 300 pN for retraction speeds of 70 μm/s when the distance between cantilever and surface is less than 2500 nm. Making use of the expression deduced by Alcaraz et al. [51], the forces related to this hydrodynamic effect in the present work may be estimated as less than 10 pN. Analyzing the decellularized cell–matrix interaction at different speeds also offers the advantage to allow for a deeper study of the interactions established between the cell and the substrate, since it yields information on the energy landscape of this interaction [15]. However, and since the main objective of this work is the development of the described experimental procedure, a detailed analysis on this issue will be postponed to forthcoming studies.

## 5. Conclusions

The previous results and discussion illustrate the possibilities offered by single-cell force spectroscopy (SCFS) for the study of the interaction between cells and biomimetic scaffolds. As illustrated in the model system formed by porcine adipose-derived mesenchymal cells and decellularized cardiac patches, SCFS allows obtaining results in a very short time and with higher precision than those recorded with other more conventional techniques employed to characterize this interaction. Specifically, in this work SCFS is applied to the validation of an assessment procedure intended to allow the development of enhanced cardiac patches. A comprehensive SCFS methodology is presented, which may allow the evaluation of systems composed by different combinations of cell lineages and scaffolds by quantitatively determining the involved adhesion forces. The measurement of these adhesion forces, in turn, offers a guide for the development of new surfaces that ensure the effective retention of a sufficient number of cells. This way the intended therapeutic effect of this approach, as expected from cardiac patches, can be achieved.

## Data Availability

The original data presented in the study are included in figures throughout the article. Further reasonable inquiries regarding data access can be directed to the corresponding authors.
